# In Vitro and In Vivo Activity of Luliconazole (NND-502) against Planktonic Cells and Biofilms of Azole Resistant *Aspergillus fumigatus*

**DOI:** 10.3390/jof8040350

**Published:** 2022-03-28

**Authors:** Dan-Tiberiu Furnica, Silke Dittmer, Maike Isabell Sanders, Joerg Steinmann, Peter-Michael Rath, Lisa Kirchhoff

**Affiliations:** 1 Institute of Medical Microbiology, University Hospital Essen, University of Duisburg-Essen, 45122 Essen, Germany; silke.dittmer@uk-essen.de (S.D.); maike.sanders@posteo.de (M.I.S.); joerg.steinmann@klinikum-nuernberg.de (J.S.); peter-michael.rath@uk-essen.de (P.-M.R.); lisa.kirchhoff@uk-essen.de (L.K.); 2 Institute of Clinical Hygiene, Medical Microbiology and Infectiology, Klinikum Nürnberg, Paracelsus Medical University, 90419 Nuremberg, Germany

**Keywords:** ARAF, luliconazole, biofilm, *Galleria mellonella*

## Abstract

*Aspergillus fumigatus* has become a significant threat in clinical settings. Cases of invasive infections with azole-resistant *A. fumigatus* isolates (ARAF) increased recently. Developing strategies for dealing with ARAF has become crucial. We here investigated the in-vitro and in-vivo activity of the imidazole luliconazole (LLCZ) against clinical ARAF. In total, the LLCZ minimum inhibitory concentrations (MICs) were tested for 101 *A. fumigatus* isolates (84 ARAF and 17 azole-susceptible *A. fumigatus* as wild-type controls) according to the European Committee on Antimicrobial Susceptibility Testing (EUCAST). Additionally, antifungal activity was assessed in vitro, including an XTT planktonic growth kinetics assay and biofilm assays (crystal violet and XTT assay). Further, a single-dose LLCZ treatment (152 mg/L) was tested for seven days in vivo in a *Galleria mellonella* infection model. LLCZ showed an MIC_50_ of 0.002 mg/L and no significant difference was found between triazole-resistant and wild-type isolates. Growth inhibition took place between 6 and 12 h after the start of incubation. LLCZ inhibited biofilm formation when added in the pre-adhesion stages. In vivo, single-dose LLCZ-treated larvae show a significantly higher survival percentage than the control group (20%). In conclusion, LLCZ has activity against planktonic cells and early biofilms of ARAF.

## 1. Introduction

*Aspergillus fumigatus* is among the most frequently isolated fungi in invasive mold diseases. *A. fumigatus* is a saprotrophic filamentous fungus, often described as an airborne opportunistic pathogen [1]. It is known to be responsible for several diseases, including allergic or invasive aspergillosis, especially in immunocompromised patients [2]. With increasing numbers of immunocompromised patients, the incidence of invasive aspergillosis rose over the last years [3]. Azole-resistant *A. fumigatus* (ARAF) has only made this problem worse, with some isolates being capable of mutations that help them evade the effects of triazoles [4]. Triazoles act by targeting the 14 α demethylase (coded by the *cyp51A* gene), an important enzyme in the ergosterol synthesis pathway. Through point mutations and a tandem repeat promoter alteration in the *cyp51A* gene, ARAFs manage to evade this mechanism [5]. The most frequently detected mutations are TR34/L98H followed by TR46 mutations, which were identified in both clinical isolates and environmental probes worldwide [6]. Another important factor that increases the fungal resistance to triazoles is the formation of biofilms [7]. Biofilms help the pathogens evade the hosts’ immune system, which is a major cause of concern, especially in the case of patients with reduced immunocompetence [8]. Strategies for resistance surveillance and testing are in place and the development of alternative strategies against triazole-resistant pathogens is necessary [9,10]. Today LLCZ is commercially available as a cream for therapy of *tinea pedis* (athlete’s foot) [11], as of 2015, the food and drug administration (FDA) has approved LLCZ for topic use in the United States [12]. No systemic formulation of the drug is yet available on the market. An animal study showed that the lethal dose for LLCZ is 2000 mg/kg in rats, which would indicate a potentially safe pharmacokinetic profile of the drug [13]. Clinical studies are necessary to assess the safety profile of LLCZ for an oral application in humans. While in vitro studies on LLCZ activity against *Aspergillus* spp. have been reported [14,15,16,17], data on LLCZ antifungal activity against biofilms and in vivo, especially against ARAF, is lacking. The aim of this study was to show LLCZ activity against planktonic ARAF and ARAF biofilm formation in vitro as well as to show the pathogenicity inhibiting effect against ARAF isolates in vivo.

## 2. Materials and Methods

### 2.1. Isolates

The study includes a total of 101 *A. fumigatus* isolates, which were identified through fungal ITS sequencing [18]. The isolates were either azole-resistant (*n* = 84 with different mutations of the *cyp51A* gene) or non-resistant (*n* = 17 wild type controls). Ten of these isolates were further used for the growth kinetics assay, biofilm assay, and in vivo assay. Of these, seven isolates carried a TR34/L98H mutation, one carried a TR46 mutation and two were wild-type isolates. All isolates used in this study and their specific mutations are summarized in Table 1.

### 2.2. Preparation of Fungal Suspensions

*A. fumigatus* isolates were grown on Sabouraud agar (Oxoid, Wesel, Germany) for 2 days at 35 °C. An H_2_O + 0.1% Tween solution was added to the plates, the fungal suspension was collected with a syringe and filtered (10 μm pore size, syringe Filcons, BD, Franklin Lakes, NJ, USA). After two washing steps with 1 × phosphate-buffered saline (PBS), the inoculum was set to an appropriate concentration, via dilution in RPMI 1640, depending on the experiment. Colony-forming units (CFU) were determined as an inoculum control.

### 2.3. Preparation of LLCZ

LLCZ was purchased from Sigma-Aldrich (St. Louis, MO, USA). The drug was diluted in DMSO and a stock solution was stored until use at −20 °C. For use, the stock was diluted to working concentrations in RPMI 1640 medium. For further information on concentrations and dilutions, refer to the corresponding sections below.

### 2.4. In Vitro Experiments

#### 2.4.1. Broth Microdilution

To determine the specific minimal inhibitory concentration (MIC) of LLCZ, the broth microdilution according to the European Committee on Antimicrobial Susceptibility Testing (EUCAST) was performed [19]. LLCZ was diluted in DMSO and double concentrated RPMI 1640 medium (0.2% glucose). LLCZ concentrations ranged between 0.0004–0.25 mg/L.

#### 2.4.2. Growth Kinetics

A colorimetric micro-broth kinetic growth assay was performed to assess the impact of LLCZ on the growth of the above-mentioned isolates [20]. The isolate-specific MIC and concentrations 4- and 16-fold higher than the determined MIC were used. A growth control without LLCZ was performed in addition. The fungal inoculum (2.5 × 10^5^ CFU/mL) was incubated together with the different concentrations of LLCZ in flat-bottomed 96-well plates at 35 °C. The growth was assessed through OD_492_ measurement after 0 h, 6 h, 12 h, 24 h, and 48 h. Two hours prior to the end of incubation time, 50 µL of 2.5 mg/mL XTT (Santa Cruz Biotechnology, Dallas, TX, USA) plus 125 µM menadione (Sigma) solution were added to each well. A total of 150 µL of the suspensions were transferred to new 96-well plates with a U-shaped bottom. The absorbance for each well was read at 492 nm with a microplate reader (Sunrise, Tecan, Switzerland).

#### 2.4.3. Biofilm Formation & Biofilm Susceptibility Assays

Biofilm formation has been assessed as described previously [21]. A final inoculum of 2.5 × 10^5^ CFU/mL was used for the experiments. To assess LLCZ anti-biofilm activity, the agent was used in doses equal to the previously determined isolate-specific MICs, as well as in sub-MIC concentrations: a half, a quarter, and an eighth of the specific MIC value. A negative and a non-treated control were also included in this experiment. The drugs were added at different time points after incubation start at 35 °C: 0 h, 4 h, 12 h, 24 h, and 48 h. The plates were incubated at 35 °C for biofilm development. After incubation, they were washed with 1 × PBS and 200 µL LLCZ was added. The biofilm was incubated with LLCZ for a further 24 h at 35 °C. Afterward, it was washed twice with 1 × PBS and analyzed via XTT assay. Therefore, 100 µL/well of an XTT (0.5 mg/mL) plus 25 µM menadione solution were pipetted onto the plates and incubated at 36 °C for 3 h. The optical density was measured in a plate reader at 492 nm.

#### 2.4.4. Confocal Laser Scanning Microscopy (CLSM)

The anti-biofilm effect was visualized in a confocal laser scanning microscopy CLSM using the ELYRA LSM 710 (Zeiss, Oberkochen, Germany) with a laser at 405 nm and a 20-fold magnification objective. Fungal suspensions were incubated and treated as described above in 1 µ-Slide 8-well glass-bottom dishes (ibidi GmbH^®^, Gräfelfing, Germany). The formed biofilms were fixed with 100% methanol for 1 min. Images were processed using the ZEN black software (Zeiss) [21]. 

### 2.5. In Vivo Experiments

#### 2.5.1. Handling of Galleria Mellonella Larvae

The larval stage of the greater wax moth (*G. mellonella*) was chosen for in vivo experiments. The protocols for handling the larvae were previously optimized and published [22]. In brief, the larvae were separated into groups, according to their weight. Injection of the drugs and fungi was performed with a syringe pump (SyringePumpPro model LA-100; Landgraph Laborsysteme HLL GmbH, Langenhagen, Germany), with the injected volume corresponding to the larval weight (10 µL for 300 ± 50 mg larvae). Larvae were incubated over seven days at 37 °C and survival was monitored daily.

#### 2.5.2. Treatment Assay

This assay tested the effect of a single LLCZ dose against an infection with *A. fumigatus* in *G. mellonella*. Three *A. fumigatus* isolates were used for this experiment (two ARAFs and a wild type, Table 1). An inoculum of 2.5 × 10^5^ CFU/mL [23] was injected into the larvae, based on data from an infection assay (Figures S2 and S3). LLCZ was applied in concentrations of 5 mg/kg body weight, equaling 152 mg/L [17]. The larvae were infected and incubated as described above and the LLCZ treatment was performed 24 h after infection, in a different pro-leg, in order to avoid any excessive stress of the larvae. The survival was monitored for 7 days. Three control groups were used: injection on two different days with PBS, injection with fungal inoculum, injection with PBS as well as a non-injection control. 

#### 2.5.3. Re-Cultivation of *A. fumigatus*

The re-cultivation of dead larvae was performed as previously described [22]. Upon disintegration using the MagNa Lyser (Roche, Grenzach-Wyhlen, Germany), larvae were plated on Sabouraud agar to confirm the re-isolation of the pathogen.

### 2.6. Statistical Analysis

#### 2.6.1. In Vitro

All experiments were performed in triplicates. The growth percentage in every treated well was calculated in report to the untreated control. A statistical analysis of the growth percentage was carried out with the program GraphPad Prism 9 (GraphPad Software Inc., La Jolla, CA, USA). The statistical significance level was determined by Dunnett’s multiple comparison test, *p* < 0.05 was stated as significant and the confidence score was indicated by asterisks: *: *p* < 0.05, **: *p* < 0.01, ***: *p* < 0.001, ****: *p* < 0.0001. 

#### 2.6.2. In Vivo

All experiments were performed in triplicates. The survival data were analyzed with the program GraphPad Prism 9 (GraphPad Software Inc.). Survival curves were plotted with the Kaplan-Meier estimator. To compile results from multiple repetitions, the raw data was calculated as a single experiment on the datasheet. The statistical significance of the survival curves was determined with a log-rank (Mantel-Cox) test. *p* < 0.05 was stated as significant and the confidence score was indicated by asterisks: *: *p* < 0.05, **: *p* < 0.01, ***: *p* < 0.001, ****: *p* < 0.0001.

## 3. Results

**Figure 1:**
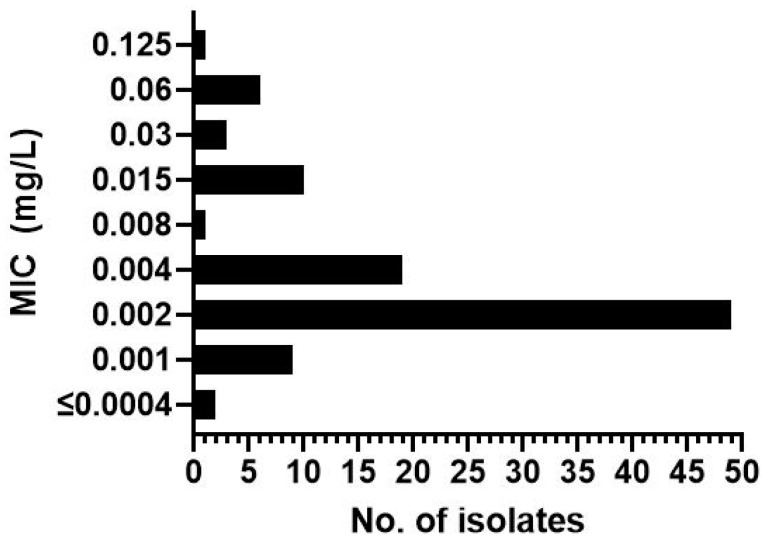
Minimum inhibitory concentrations (MIC) in mg/L for LLCZ against 101 *A. fumigatus* isolates, 84 of which were triazole resistant.

The MIC values of LLCZ ranged between ≤0.0004 mg/L and 0.125 mg/L. The overall MIC_50_ value was 0.002 mg/L and the MIC_90_ value was 0.015 mg/L. No significant differences in the MIC values could be noticed between mutants and wild-type isolates (Table 1 and Figure 1).

**Figure 2:**
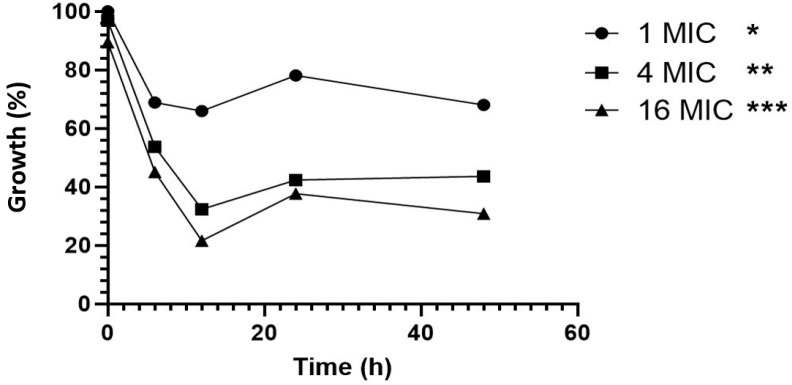
Growth kinetics of 10 *A. fumigatus* isolates with a percentual representation in report to the untreated control. Isolates were treated with LLCZ concentrations four times and 16 times the isolate specific MIC. Incubation took place at 35 °C for 48 h. The optical density was measured at 492 nm after 2 h, 6 h, 12 h, 24 h, and 38 h of incubation. Metabolically active cells were determined by incubation with 50 µL XTT (2.5 mg/mL) + menadione (125 mg/mL) per well (the XTT was added 2 h prior to incubation end). Statistical significance was determined by Dunnett’s multiple comparison tests, *p* < 0.05 was stated as significant and the confidence score was indicated by asterisks: *: *p* < 0.05, **: *p* < 0.01, ***: *p* < 0.001.

LLCZ activity against ARAF planktonic growth over time was determined via an XTT growth kinetic assay. The different LLCZ concentrations had a significant effect on the growth of the 10 tested isolates, with an inhibitory effect already being visible between 6 and 12 h after incubation with the drug. After 12 h of incubation, the isolates seemed to be able to fully (1 × MIC) or partially (4 × and 16 × MIC) recover from the effect of the drug (Figure 2).

**Figure 3:**
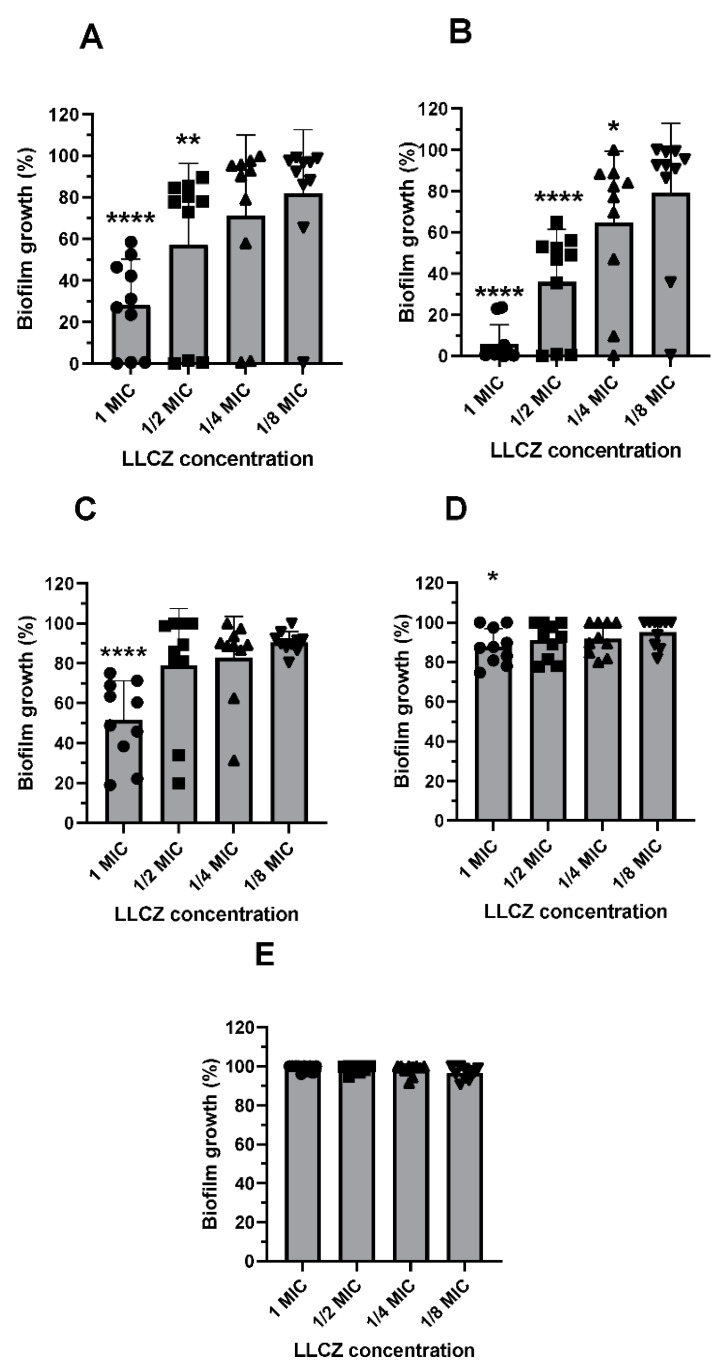
Growth of *A. fumigatus* biofilm (10 isolates), when treated with luliconazole (XTT assay). Isolates were treated with concentrations of 1- (●), 1/2- (■), 1/4- (▲) and 1/8 (▼) times the isolate specific minimal inhibitory concentration at: (**A**). 0 h (**B**). 4 h, (**C**). 12 h (**D**). 24 h and (**E**). 48 h after incubation start at 35 °C. The isolates were incubated with LLCZ for 24 h at 35 °C and subsequently washed twice with 1 × PBS and incubated with XTT (0.5 mg/mL) + Menadione (25 µM) for 3 h. The optical density was measured at 492 nm. Statistical significance was determined by Dunnett’s multiple comparison tests, *p* < 0.05 was stated as significant and the confidence score was indicated by asterisks: *: *p* < 0.05, **: *p* < 0.01, ****: *p* < 0.0001.

**Figure 4:**
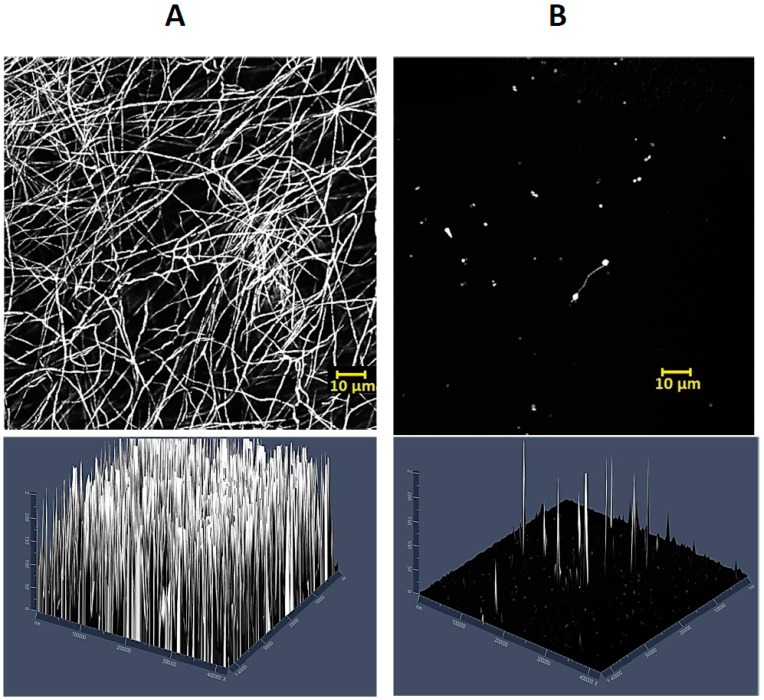
CLSM 2 D (top) and 2.5 D (bottom) images of ARAF (isolate 1954) biofilm. (**A**) Untreated; (**B**) treated with one-half the isolate specific MIC (0.002 mg/L) 4 h after incubation start.

Afterwards, the effect of LLCZ on ARAF biofilm formation was tested via XTT and CV assay and visualized via CLSM: Both, crystal violet and XTT assay indicated that the best biofilm growth inhibition occurred when LLCZ was added 4 h after incubation start, in the pre-adhesion phase. On the more mature biofilm (12 h, 24 h, or 48 h after incubation start), LLCZ treatment was less effective or had no effect at all (Figure 3 and Figure S1). Upon visualizing the biofilm, CLSM showed a decrease in biofilm mass depending on the applied LLCZ concentration. One-half of the isolate-specific MIC (0.002 mg/L) was enough to fully inhibit the biofilm formation of an ARAF isolate when applied 4 h after incubation start at 35 °C (Figure 4).

**Figure 5:**
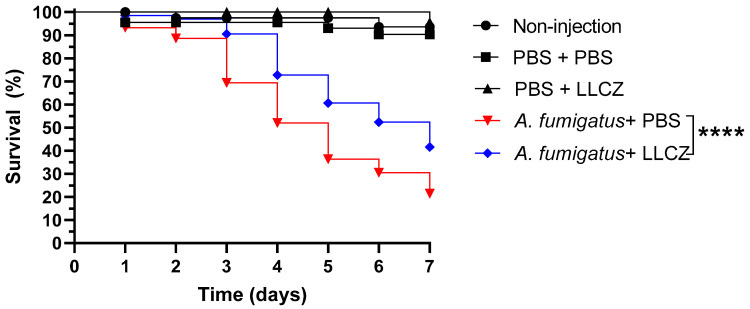
Survival curves for Galleria mellonella larvae treated with placebo (PBS) and LLCZ during a seven-day period. Groups of 15 larvae were injected with a fungal inoculum of 2.5 × 10^5^ CFU/mL and then after 24 h with 152 mg/L LLCZ (treatment) or PBS (placebo). Control groups were initially injected with PBS and with either PBS or LLCZ on the second day, in order to rule out any false-positive results caused by the piercing damage of the needle or by LLCZ. A non-injection control was used as an indicator for the quality of the larvae. The larvae were incubated at 37 °C and mortality was checked every 24 h for at least 7 days. Results shown are the average values for all of the three isolates (1954, 2107, and the wild type). Statistical significance was determined by Dunnett’s multiple comparison tests. ****: *p* < 0.0001.

LLCZ showed no toxicity in the in vivo experiment model (Figures S2 and S3). An infection assay showed that there was no visible difference between the three isolates regarding infection speed, virulence, or lethality, all larvae groups having a survival outcome of approximately 10–20% after 7 days. Most larvae started to die 3–4 days after infection with the number of dead larvae depending on the concentration of the inoculum (Figures S2 and S3 ). No significant differences were found between the different inoculum concentrations and isolates. In the treatment experiments, larvae that were infected with an inoculum of 2.5 × 10^5^ cells/mL and treated with a therapeutic dose of 152 mg/L LLCZ, had a survival percentage of 41% over the course of 7 days. The larvae treated with a placebo (PBS) had a survival outcome of 21%, similar to the result of the infection assay (Figure 5).

## 4. Discussion

This study provides in vitro data for the planktonic growth kinetics and biofilm development of ARAF treated with LLCZ. Moreover, it also proved the efficiency of LLCZ against ARAF infections using a non-vertebrate in vivo model. 

This study, with 101 isolates tested according to EUCAST, showed that the MIC values for LLCZ ranged between ≤0.0004 mg/L and 0.125 mg/L, with no significant differences being observed between the triazole-resistant mutants and wild type isolates. These results were similar to those of Abastabar et al. [15], despite them using a different MIC determination method after The Clinical and Laboratory Standards Institute (CLSI). Recent publications studied the effect of LLCZ against *A. fumigatus* and recognized the possible benefits that this drug could have in a systemic formulation [11,15]. Except for its remarkable effects against *A. fumigatus*, LLCZ is also known to have a significant effect against black aspergilli such as *A. niger*, *A. tubigensis,* and *A. piperis* [14].

The effect of LLCZ against planktonic ARAF growth was determined via an XTT assay where the metabolic activity of the treated cells was detected and compared to that of the untreated cells at different time points. As far as we know, this is the first study that showed the growth kinetics of planktonic ARAF treated with LLCZ. Results show that the drug was effective between 6 h and 12 h of incubation with the cells. In the *A. fumigatus* development cycle, these time points correspond to the stage where the conidial germ tubes emerge and extend. However, this precedes the hyphal development stage [24]. The metabolic activity slowly recovers between 12 h and 48 h. Since the antifungal agent was not able to reduce 99% of the fungal load, it was characterized as a fungistatic compound [25]. A possible explanation for this phenomenon could be that after 12 h of incubation with LLCZ, the cells developed a biofilm that increased their resistance towards LLCZ. Therefore, the next in vitro experiment analyzed the activity of LLCZ against ARAF biofilm in different development stages.

Treatment of biofilm-based infections has become a major challenge in clinical settings, with only a few antifungal agents still being effective against mature biofilm [26]. Treatment becomes twice as difficult if the pathogens possess triazole resistance. Two examples of drugs that still have an effect against biofilms would be amphotericin B and echinocandins such as micafungin [27]. While the anti-biofilm effect of LLCZ has been assessed against *Rhodotorula* spp. with no significant inhibition being detected [16], so far no data is available on the effect of LLCZ against wild type *A. fumigatus* or ARAF biofilm. We here detected a significant difference of about 20% between the 0 h and 4 h time points, with more biofilm growth at 0 h. An explanation for this could be that when the antifungal was added at 4 h, 12 h, 24 h, and 48 h, the plates were rinsed once with PBS. It is possible that a small part of the still viable conidia was washed away during this process. The washing step could account for the 10–20% loss in biofilm mass and for the difference between 0 h and 4 h. LLCZ seemed to be less effective when added at 12 h, 24 h and 48 h. A concentration of one time the MIC managed to reduce only half of the biofilm growth when added 12 h after incubation start, while the sub-MIC concentrations had no significant effect in biofilm reduction. LLCZ was less active against 24 h biofilms and had no effect against 48 h old biofilms. Similarly, the triazole antifungal intraconazole has been described to have a comparable effect against the biofilms of wild type *A. fumigatus* isolates [28]. It has been shown that the novel antifungal drug olorofim has a comparable antibiofilm activity against the early stages of biofilm formation and only a reduced effect against mature biofilm [21].

Most information related to the effects of LLCZ is based on in vitro studies of clinical *A. fumigatus* isolates and only limited data is available on in vivo assays. Previous in vivo experiments have been performed on rats, however, the therapeutic effect of LLCZ has only been tested against wild type *A. fumigatus* strains [17]. Until now, it remained unclear if this antifungal agent had an effect against ARAF in vivo.

We here demonstrated the effect of LLCZ treatment in an in vivo model. Because of the high availability of larvae and their short life cycle, *G. mellonella* is a viable choice for the initial testing of an LLCZ systemic treatment against ARAF [22]. Preliminary testing showed the low toxicity of the drug in the invertebrate animal model (Figures S2 and S3) and proved the lethality of *A. fumigatus* isolates when injected. Treatment assays showed a 20% increase in the survival of the treated LLCZ larvae in comparison to the larvae treated with PBS. Despite the fact that the 7-days survival percentage of the treated larvae was lower than 50%, it must be mentioned that the applied therapeutic dose was adapted from an in vivo rat study, where this dosage was applied daily [17]. Even though *G. mellonella* is a good model with multiple advantages such as extensive testing of drug toxicity and distribution, it has several drawbacks, one of them being the physiological difference to the human model [29]. However, it is a suitable model for designing a therapy strategy with a less-studied drug such as LLCZ and it surpassed other invertebrate animal models such as *Caenorhabditis elegans* where the drug cannot be directly injected into the host organism [22]. 

For future analysis, it is suggested to enlarge the number of isolates included in both in vitro biofilm and in vivo assays to prove the here-demonstrated activity of LLCZ. 

## 5. Conclusions

In conclusion, we were able to demonstrate LLCZ activity against *A. fumigatus* in vitro and in vivo. LLCZ showed an inhibitive effect against the planktonic growth of *A. fumigatus*, regardless of the presence of *cyp51a* mutations. It showed relatively low MIC values ranging between ≤0.0004 mg/L to 0.125 mg/L, an MIC_50_ value of 0.002 mg/L and an MIC_90_ value of 0.015 mg/L. It inhibited planktonic growth between 6–12 h after incubation start and the metabolic activity of the cells slowly recovered between 12 and 48 h. The activity of LLCZ against biofilm formation could be correlated to this regenerative effect, since it was demonstrated that the drug disrupts early-stage biofilm formation (0 h and 4 h) but is inefficient against mature biofilm (24 h and 48 h). In vivo experiments showed one injection of LLCZ could significantly improve *G. mellonella* survival after infection with ARAF. Considering the above-mentioned results and the evident in vitro and in vivo activity of LLCZ against ARAF growth and biofilm formation, this drug might be considered as an alternative for a future systemic treatment of ARAF.

## Figures and Tables

**Table 1:** List of all the *A. fumigatus* isolates used in this study, with isolate specific MIC (µg/mL) values of LLCZ and their characteristic mutations. Strains marked with an asterisk * were used in subsequent assays.

Isolate	MIC (mg/L)	Mutation	Isolate	MIC (mg/L)	Mutation	Isolate	MIC (mg/L)	Mutation	Isolate	MIC (mg/L)	Mutation
1954 *	0.002	TR34/L98H	1962	0.002	TR34	2120	0.06	TR34	2320	0.015	Wild type
2107 *	0.06	TR46	1964	0.001	TR34	2122	0.002	TR34	2321	0.015	Wild type
2514 *	0.002	TR34/L98H	1966	0.002	TR34	2123	0.004	TR34	2325	0.03	Wild type
2607 *	0.002	TR34/L98H	1970	0.001	TR34	2130	0.002	TR34	2328	0.002	Wild type
2608 *	0.002	TR34/L98H	1972	0.002	TR34	2142	0.002	TR34	2331	0.015	TR34/L98H
2135 *	0.03	TR34/L98H	1974	0.001	TR34	2147	0.002	Wild type	2332	0.004	TR34/L98H
2515 *	0.002	TR34/L98H	1975	≤0.0004	Wild type	2166	0.002	TR34/L98H	2333	0.015	TR34/L98H
2119 *	0.002	TR34/L98H	1976	0.004	TR34	2173	0.002	TR34	2334	0.015	Wild type
m1215 *	0.002	Wild-type	1978	0.002	TR34	2199	0.002	TR34	2344	0.015	Wild type
ATCC 204305 *	0.002	Wild-type	1979	0.004	Wild type	2204	0.002	TR34	2346	0.06	TR34
1944	0.004	TR34/L98H	1984	0.004	TR34/L98H	2246	0.002	TR34	2347	0.015	TR34/L98H
1965	0.004	TR34	1986	0.002	TR34	2249	0.002	TR34	2348	0.06	TR34/L98H
1968	0.004	TR34	2010	0.004	TR34	2250	0.002	TR34	2688	0.002	TR34
1971	0.002	TR34	2026	0.002	TR34	2258	0.002	TR34	2689	0.002	TR34
2102	0.004	TR34	2035	0.002	Wild type	2259	0.002	TR34	2690	0.03	TR34
2133	0.002	TR34	2036	0.001	Wild type	2300	0.004	TR34/L98H	2691	0.008	TR34
2136	0.004	TR34	2039	0.001	TR34	2310	0.004	TR34/L98H	2692	0.001	Wild type
2198	0.009	TR34	2040	0.001	TR34	2311	0.002	TR34/L98H	2693	0.002	TR34
2237	0.004	TR34	2044	0.004	TR34	2312	0.004	TR34/L98H	2694	0.06	TR34
2513	0.001	TR34/L98H	2047	0.002	TR34	2313	0.002	TR34/L98H	2702	0.002	TR34
2453	0.125	TR46	2087	0.002	TR34	2314	0.001	TR34/L98H	2707	0.002	TR34
2487	0.06	TR46	2089	0.002	TR34	2315	0.004	TR34/L98H	2708	0.004	TR34/L98H
1950	0.002	TR34	2091	0.002	TR34	2316	0.015	TR34/L98H	2711	0.002	TR34
1958	≤0.0004	Wild type	2101	0.004	TR34	2317	0.015	TR34/L98H	2712	0.002	TR34
1959	0.002	TR34	2116	0.002	TR34	2319	0.015	Wild type	2713	0.002	Wild type
									2714	0.002	TR34

## Data Availability

The data presented in this study are available on request from the corresponding author.

## Supplementary Materials

The following supporting information can be downloaded at: https://www.mdpi.com/article/10.3390/jof8040350/s1, Figure S1: Growth of *A. fumigatus* biofilm (10 strains), when treated with luliconazole (CV stain). Figure S2: Survival curves for *Galleria mellonella* larvae infected with. Figure S3: Survival curves for *Galleria mellonella* injected with.
