# Supplementary material for: In Vitro and In Vivo Activity of Luliconazole (NND-502) against Planktonic Cells and Biofilms of Azole Resistant Aspergillus fumigatus
Source: J Fungi (Basel). 2022 Mar 28;8(4):350. doi: 10.3390/jof8040350 (PMC9025574; doi:10.3390/jof8040350)
Supplement: Supplementary file 1 [file jof-08-00350-s001.zip › Figure S1.pdf]

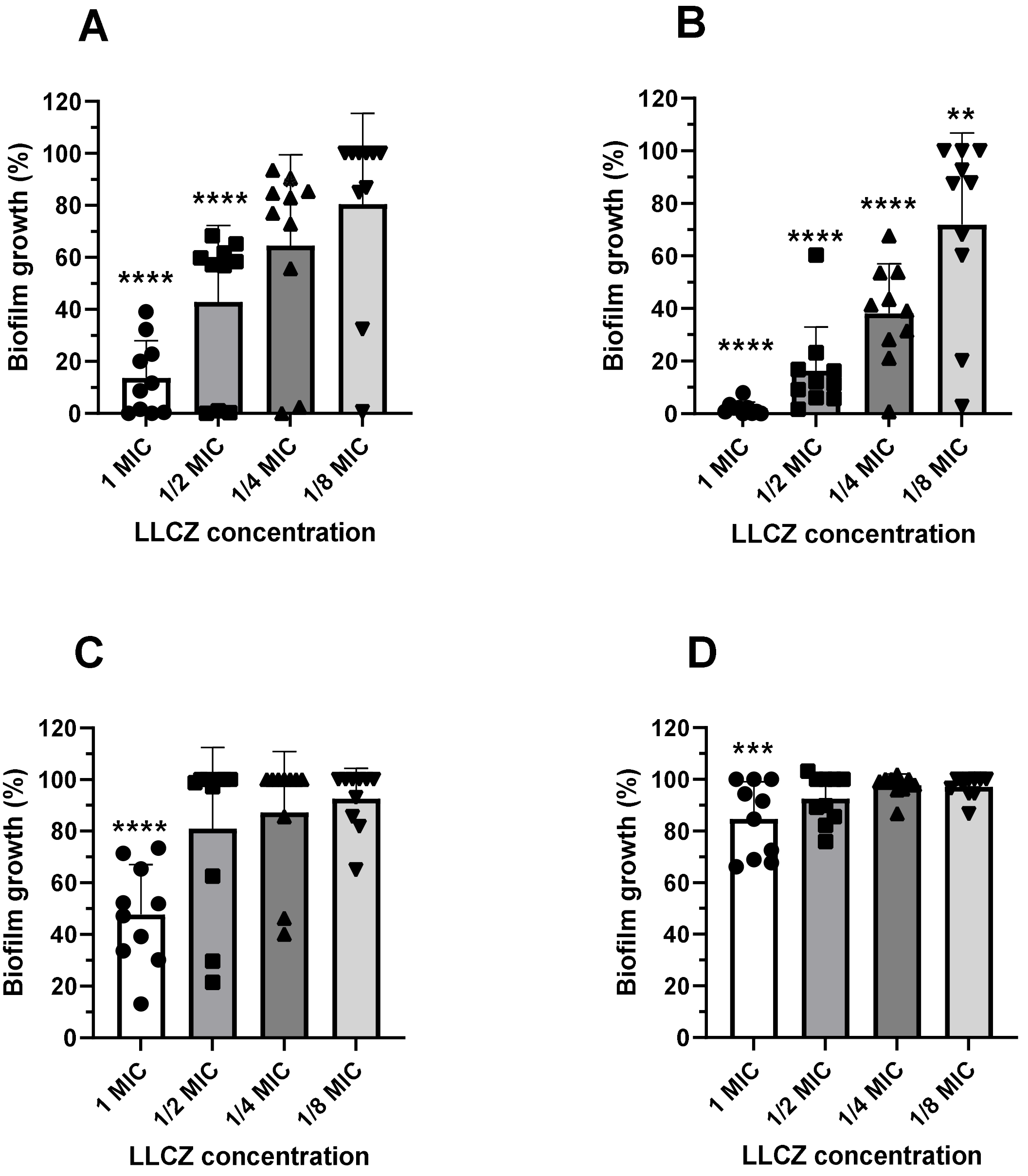


**Figure S1.** Growth of *A. fumigatus* biofilm (10 strains), when treated with luliconazole (CV assay). Concentra tions of 1-, 1/2-, 1/4- and 1/8 times the strain specific minimal inhibitory were used at: A. 0h after incubation start, B. 4 h after incubation start, C. 12 h after incubation start and D. 24 h after incubation start. The strains were incubated with the drug for 24 h at 35 °C and subsequently washed twice with 1 x PBS, incubated in 0.01 % crystal violet solution for 20 min, washed 3 times with 1 x PBS and left to dry for 24 h. The biofilm was then incubated for 30 min with 150 µL of a 30 % acetic acid solution. The optical density was then measured at 620 nm. The experiment was done in tripple repetition. Statistical significance was determined by Dunnett's multiple comparison tests, $p < 0.05$ was stated as significant and the confidence score was indicated by asterisks:**: $p < 0.01$, ***: $p < 0.001$, ****: $p < 0.0001$.
