# Supplementary material for: In Vitro and In Vivo Activity of Luliconazole (NND-502) against Planktonic Cells and Biofilms of Azole Resistant Aspergillus fumigatus
Source: J Fungi (Basel). 2022 Mar 28;8(4):350. doi: 10.3390/jof8040350 (PMC9025574; doi:10.3390/jof8040350)
Supplement: Supplementary file 1 [file jof-08-00350-s001.zip › Figure S2.pdf]

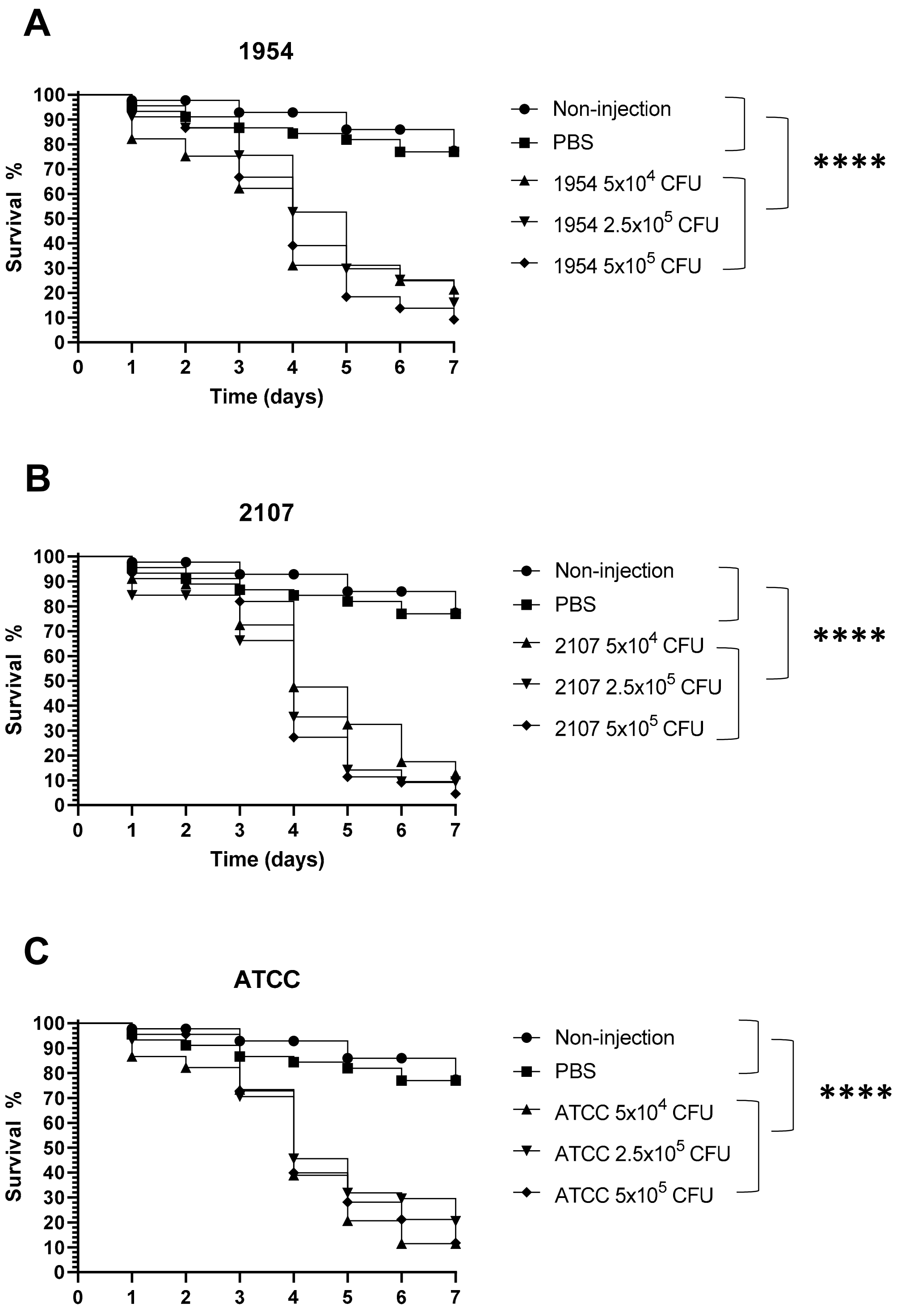

Figure S2. Survival curves for *Galleria mellonella* larvae infected with: **A**. An azole resistant *A. fumigatus* strain (strain 1954, TR34/L98H mutation), **B**. An azole resistant *A. fumigatus* strain (strain 2107, TR46 mutation) and **C**. A wildtype *A. fumigatus* strain with no mutations. Groups of 15 larvae were injected with a fungal inoculum of 5 x 10^4 CFU/ml, 2.5 x 10^5 CFU/ml and 5 x 10^5 CFU/ml, while control groups were injected with PBS or not injected at all, in order to rule out any false positive results caused by the piercing damage of the needle. The volume injected was dependent on the weight of the larvae (10-16.66 µl). The larvae were incubated at 37 °C and mortality was checked every 24 h for at least 7 days.
