# Supplementary material for: In Vitro and In Vivo Activity of Luliconazole (NND-502) against Planktonic Cells and Biofilms of Azole Resistant Aspergillus fumigatus
Source: J Fungi (Basel). 2022 Mar 28;8(4):350. doi: 10.3390/jof8040350 (PMC9025574; doi:10.3390/jof8040350)
Supplement: Supplementary file 1 [file jof-08-00350-s001.zip › Figure S3.pdf]

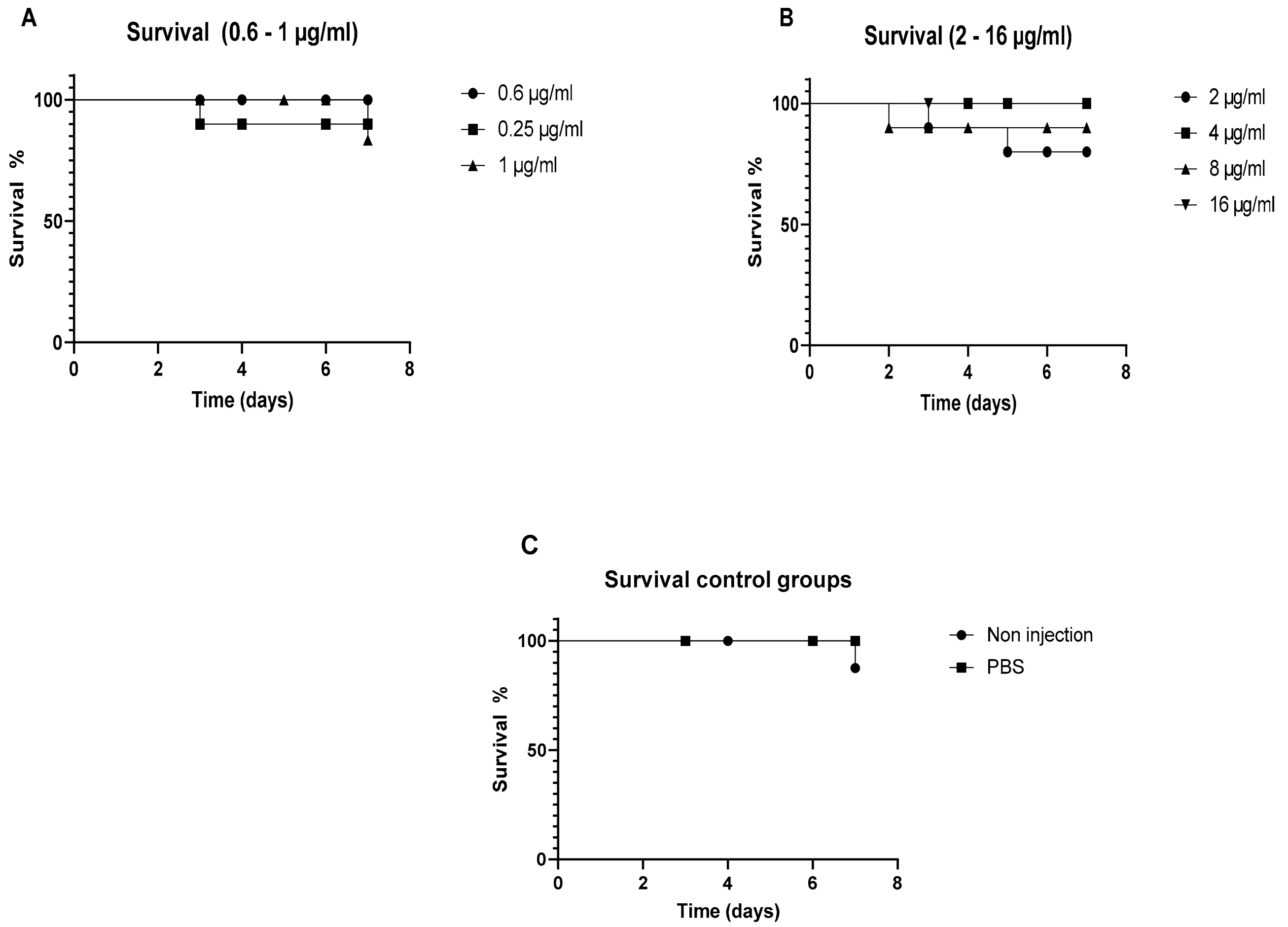

Figure S3. Survival curves for *Galleria mellonella* injected with: **A.** 0.6 - 1 µg/ml LLCZ, **B.** 2-16 µg/ml LLCZ and **C.** not injected or injected with PBS. Groups of 10 larvae were injected with the antifungal drug in different concentrations, while control groups were injected with PBS or not injected at all. The larvae were incubated at 37 °C and mortality was checked every 24 h for at least 7 days.
